# Intestinal perforation: an unusual complication of barium enema

**DOI:** 10.1590/0100-3984.2015.0222

**Published:** 2017

**Authors:** Carla Lorena Vasques Mendes de Miranda, Camila Soares Moreira de Sousa, Nathalie Gonçalves Nascimento Pinheiro Cordão, Breno Braga Bastos, Francisco Edward Mont'Alverne Filho

**Affiliations:** 1 Med Imagem – Radiologia, Teresina, PI, Brazil.; 2 UDI 24 horas – Radiologia, Teresina, PI, Brazil.

**Figure 1:**
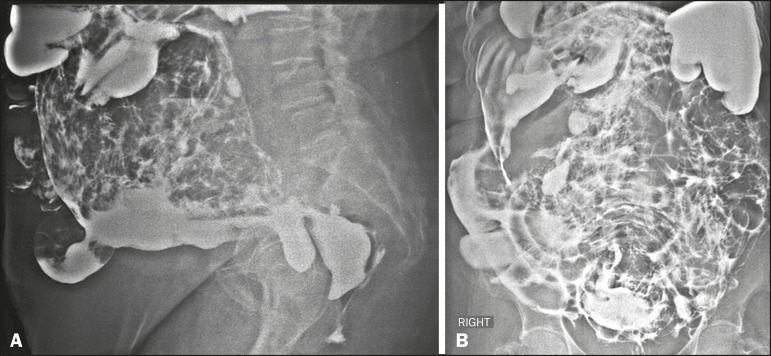
Images acquired during barium enema examination, in lateral (A) and anteroposterior (B) views.

**Figure 2:**
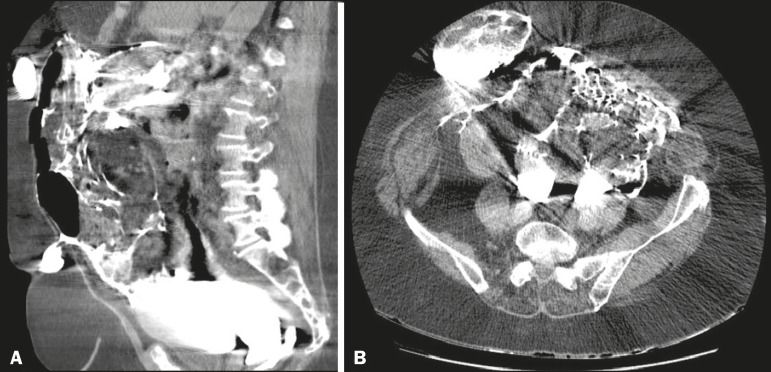
Computed tomography scans of the abdomen, in the axial (A) and sagittal (B) planes.

Dear Editor,

An 83-year-old female patient complaining of constipation was referred to our institution for elective enema with barium contrast, which showed diffuse irregularity in the mucosal folds of the colonic loops and signs of extravasation of the contrast medium into the abdomen and pelvic cavity (Figure 1). After the examination, the patient remained stable, without additional complaints. However, she did not agree to being hospitalized, signing a waiver. Despite being informed of the risks, she remained resolute, promising to return if there were any symptoms. She subsequently returned to the hospital with an acute abdomen, at which time she underwent computed tomography of the abdomen for preoperative evaluation, which demonstrated abdominal wall hernias, diverticulosis of the sigmoid colon, and a large amount of contrast material distributed diffusely throughout the peritoneal cavity and the hernias (Figure 2). The main hypothesis was perforation of the wall of the gastrointestinal tract by the enema. The patient underwent exploratory laparotomy, with an inventory of the abdominal cavity, which confirmed the tomography findings and identified a laceration at the rectosigmoid junction. After 14 days in the intensive care unit, the patient died.

Colorectal perforation is a serious complication of a barium enema. Although its exact occurrence is difficult to establish, some studies indicate a mean incidence of 0.02–0.23% among the exams performed, with a mortality rate of up to 50%^(1,2)^. The sites most commonly affected are the sigmoid colon and the rectum.

Etiologically, colorectal perforations cause by enema administration can be divided into those that are iatrogenic and those that are secondary to weakness of the colorectal wall. Iatrogenic perforations can occur as a result of forced introduction of the catheter into the anterior rectum wall, balloon hyperinflation, or excessive hydrostatic pressure during contrast injection. Perforations secondary to colorectal wall weakness occur in patients with a history of inflammatory bowel disease, acute diverticulitis, or obstructive colorectal processes, as well as in those who have recently undergone a surgical procedure, are of advanced age, or are on corticosteroid therapy, any of which make these patients more susceptible to perforation during the administration of the enema^(3)^. In such high-risk cases, the use of water-soluble contrast should be considered.

The symptoms of colorectal perforation are variable, depending on the location and size of the lesion, and can initially manifest as abdominal pain progressing to peritonitis, sepsis, and shock. However, in fewer than 10% of cases, patients are asymptomatic in the first days after the examination, and the radiologist can be the first to suggest perforation, as was the case in the patient described here^(3,4)^.

In cases of colorectal perforation in which the patient is stable, the puncture is small, and there is no fecal matter in the gastrointestinal tract or retroperitoneum, conservative treatment is adopted. Otherwise, exploratory laparotomy is necessary^(5)^.

Although barium enema is a routine examination, it should be performed with caution. In cases of perforation resulting from the examination, treatment should be initiated early and should be tailored to the type of injury, as well as to the clinical status of the patient, thus reducing the morbidity and mortality associated with the condition.
